# A case report of a blueberry muffin baby caused by congenital self-healing indeterminate cell histiocytosis

**DOI:** 10.1186/s12887-023-03922-5

**Published:** 2023-03-08

**Authors:** S. B. L. Koster, M. E. Vinke, C. van den Bos, W. J. M. van Heel, M. E. G. Kranendonk, R. Natté, A. M. van Tuyll van Serooskerken

**Affiliations:** 1 Department of Dermatology, Erasmus Medisch Centrum, Doctor Molewaterplein 40, 3015 GD Rotterdam, The Netherlands; 2 Department of Hemato-oncology, Prinses Máxima Centrum, Heidelberglaan 25, 3584 CS Utrecht, The Netherlands; 3 Department of Pediatrics, HagaZiekenhuis/Juliana Kinderziekenhuis, Els Borst-Eilersplein 275, 2545 AA Den Haag, The Netherlands; 4 Department of Pathology, Prinses Máxima Centrum, Heidelberglaan 25, 3584 CS Utrecht, The Netherlands; 5 Department of Pathology, HagaZiekenhuis, Els Borst-Eilersplein 275, 2545 AA Den Haag, The Netherlands; 6 Department of Dermatology, HagaZiekenhuis, Els Borst-Eilersplein 275, 2545 AA Den Haag, The Netherlands

**Keywords:** Blueberry muffin rash, Hashimoto-Pritzker, Indeterminate cell histiocytosis, MAP2K1

## Abstract

**Background:**

Blueberry muffin is a descriptive term for a neonate with multiple purpuric skin lesions. Many causes are known, amongst them life-threatening diseases like congenital infections or leukemia. Indeterminate cell histiocytosis (ICH) is an exceptionally rare cause of blueberry muffin rash. ICH is a histiocytic disorder which can be limited to the skin or can present with systemic involvement. A mutation that has been described in histiocytic disorders is a MAP2K1 mutation. In ICH, this mutation has previously been described in merely one case.

**Case presentation:**

A term male neonate was admitted to the neonatology ward directly after birth because of a blueberry muffin rash. ICH was diagnosed on skin biopsy. The lesions resolved spontaneously. The patient is currently 3 years old and has had no cutaneous lesions or systemic involvement so far. This disease course is similar to that of the Hashimoto-Pritzker variant of LCH.

**Conclusions:**

ICH can manifest in neonates as resolving skin lesions. It is limited to the skin in most cases, but systemic development is possible. Therefore, it is essential to confirm the diagnosis with a biopsy before the lesions resolve and to monitor these patients closely with routine follow-up.

## Background

Histiocytic disorders form a rare group of diseases caused by proliferation and accumulation of histiocytes. The term ‘histiocyte’ is used to describe immune cells from the mononuclear phagocyte system, including monocytes, macrophages and dendritic cells. Langerhans cell histiocytosis (LCH) is the most common histiocytic disorder. In LCH cells accumulate which phenotypically express Langerin (CD207), CD1a and S100. In indeterminate cell histiocytosis (ICH) the accumulating cells do not meet all these phenotypic criteria [1]. The accumulating cells express CD1a and S100 but lack Langerin. ICH was first described in 1985 and is limited to the skin in most cases. However, patients can develop systemic involvement. We present a newborn who was admitted with a blueberry muffin rash caused by ICH. A biopsy was performed to confirm the diagnosis. ICH is an exceptionally rare cause of blueberry muffin rash in neonates. In our case, the skin lesions were self-limiting, similar to the Hashimoto- Pritzker variant of LCH. This case emphasizes that a histiocytic disorder should be considered in newborns with a blueberry muffin rash. It illustrates the necessity to perform the complete work-up in time: confirm diagnosis with skin biopsy before the lesions resolve, rule out systemic involvement and arrange long-term follow-up.

## Case presentation

**Fig. 1:**
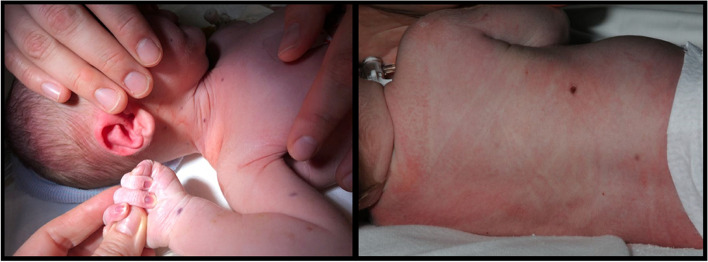
Purpura on the first day

A male neonate was born to a primigravida after in vitro fertilization. He was born at the gestational age of 39 weeks and 4 days. Apgar scores were 9 and 10 at 1 and 5 minutes. His birth weight was above the 10th percentile. There were two risk factors for infection: prelabor rupture of membranes and a group B streptococcus positive mother. Because of these risk factors, the neonate was admitted to the maternity ward for observation. In the first hour after birth, he developed purpuric skin lesions for which he was admitted to the neonatology ward. Physical examination showed several purple discolored lesions disseminated on the body which measured about 1-4 mm and did not blanch with pressure (Fig. 1). In the first couple of hours new lesions developed. The newborn looked pale but was hemodynamically stable. There was no hepatosplenomegaly.

**Fig. 2:**
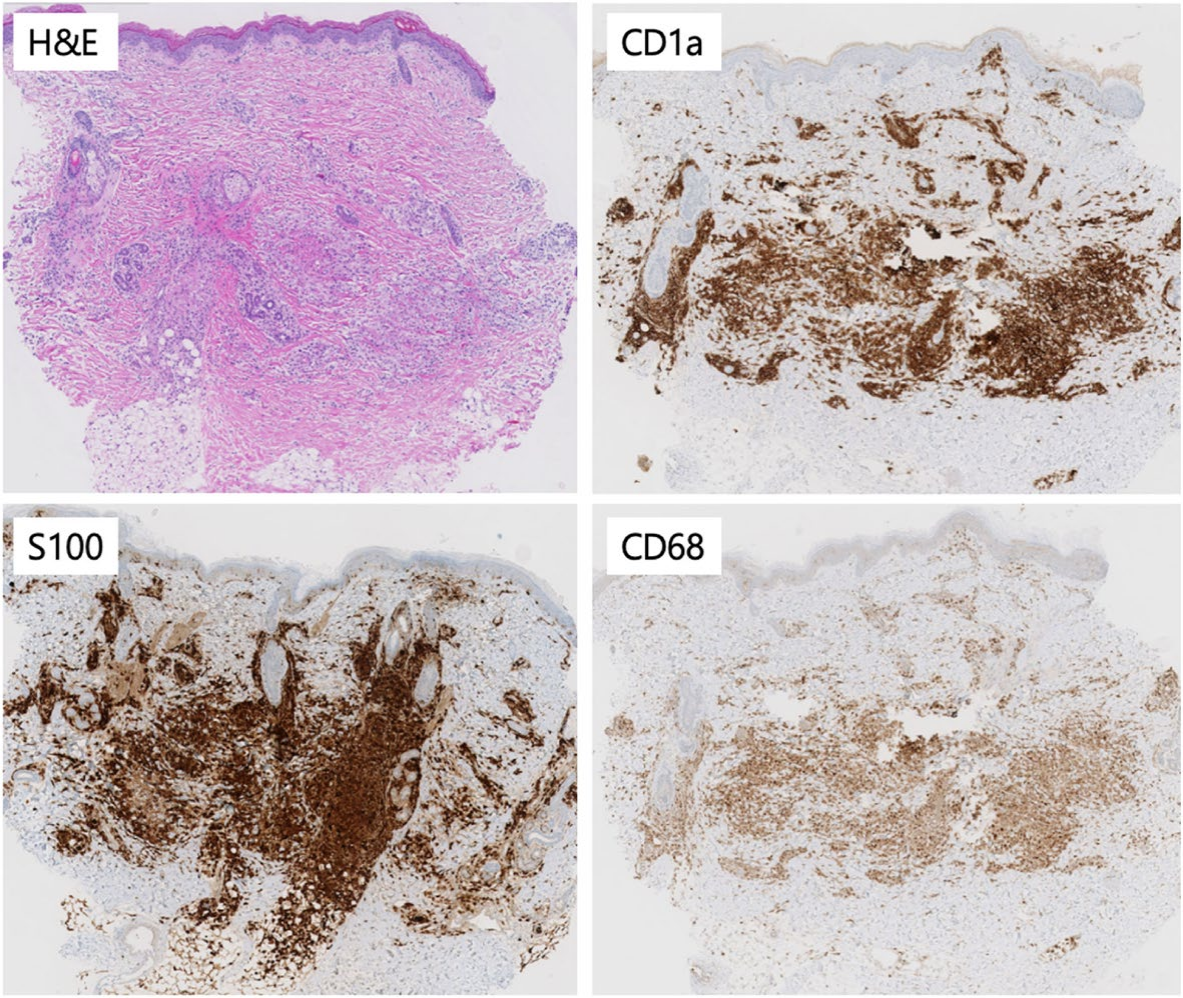
H&E: dense infiltrates in the dermis. Positive staining of lesional cells with CD1a, S100 and CD68. *All magnifications 50x*

**Fig. 3:**
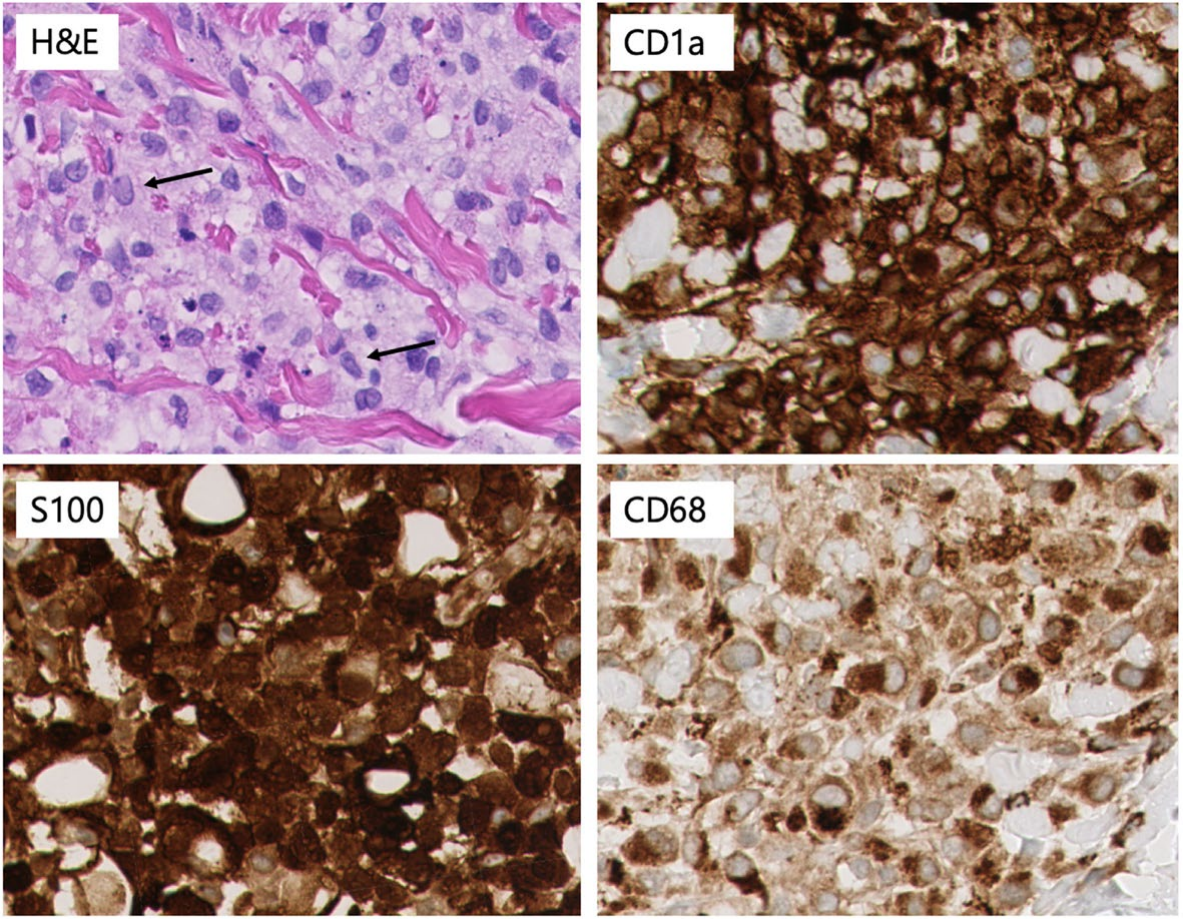
H&E: Lesional cells with abundant pale pink cytoplasm, and nuclei sometimes with nuclear groove (arrow). It is striking that there are no eosinophilic granulocytes between the tumor cells, as in an LCH. Positive staining of lesional cells with CD1a, S100 and CD68. *All magnifications 400x*

**Fig. 4:**
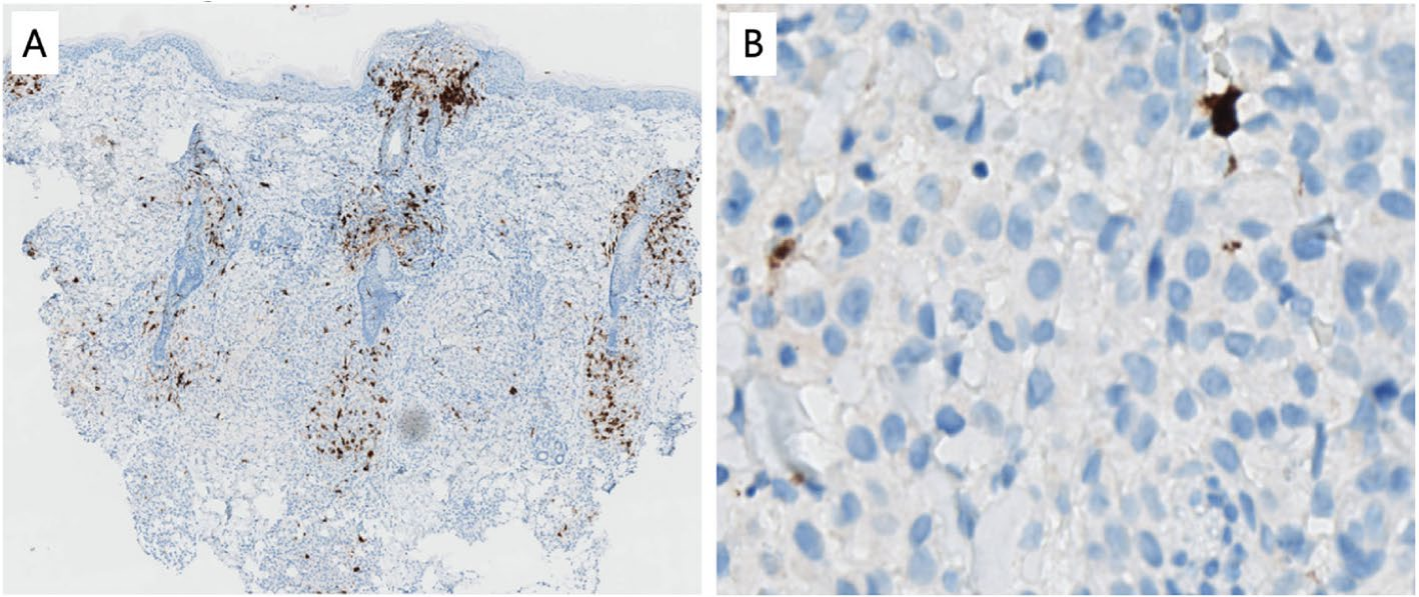
Negative staining of lesional cells with Langerin (CD207), which only stains reactive Langerhans cells. **A**: magnification 50x, **B**: magnification 400x

Laboratory examination showed no abnormalities, consisting of a full-blood count, infection markers, liver function and coagulation studies. Intravenous antibiotics were administered with the differential diagnosis of a perinatal infection. These could be stopped after 48 hours because of a good clinical condition of the newborn and a negative blood culture**.** Our differential diagnosis included a vasculitis or other dermatological condition, for which a biopsy of one of the papules was performed. The biopsy revealed dermal clusters of histiocyte-like cells with oval nuclei containing nuclear grooves and a moderate amount of eosinophilic cytoplasm. There were no eosinophilic granulocytes accompanying these lesional cells. Immunohistochemistry showed expression of CD68, CD1a and S-100 (Figs. 2 and 3), but no expression of Langerin (Fig. 4). Molecular analysis revealed a MAP2K1 mutation. Despite the resemblance with LCH, the lack of accompanying eosinophilic granulocytes and the lack of Langerin expression fits with the diagnosis ICH [2]. Ultrasonography of the brain and abdomen was normal.

**Fig. 5:**
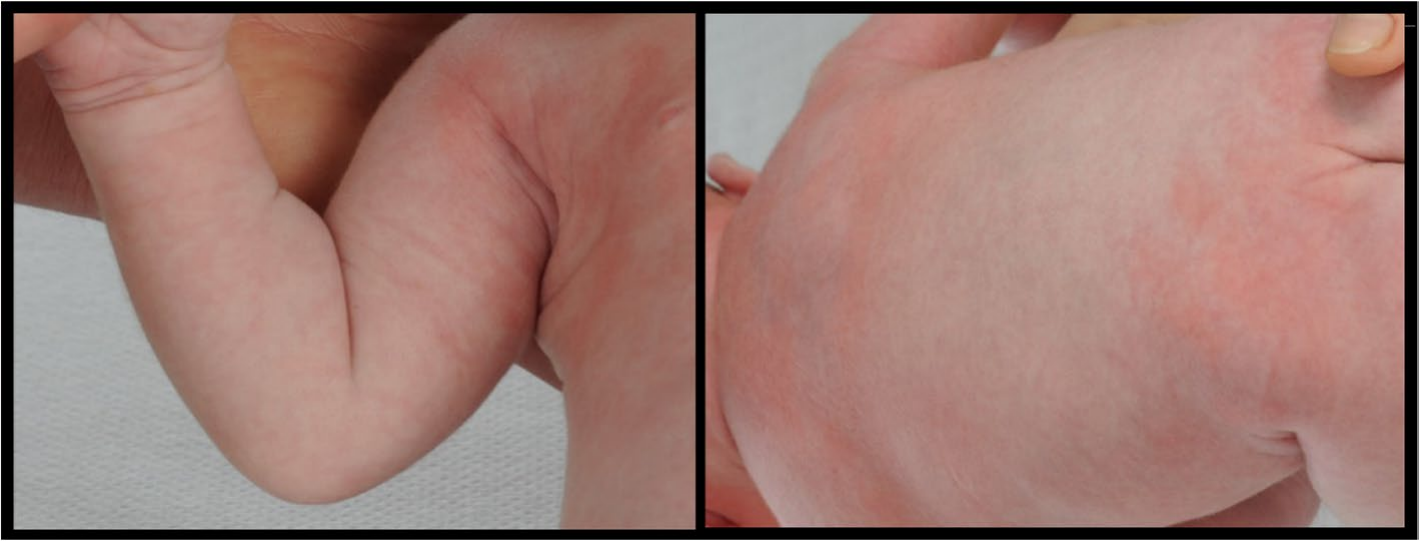
Normalisation of the cutaneous lesions 18 days after birth

Treatment of the lesions was not necessary. Five days postpartum the neonate could be discharged from the hospital. The patient was referred to a pediatric oncologist for follow-up. On his first visit to the clinic, at the age of 18 days, the cutaneous lesions had completely resolved (Fig. 5). The patient has been checked regularly for recurrence of cutaneous lesions and systemic involvement. Until now, at the age of 3 years, the patient showed no signs of recurrence. This disease course is similar to the Hashimoto- Pritzker variant of LCH.

## Discussion and conclusion

The newborn in this case presented with a blueberry muffin rash. Blueberry muffin is a descriptive term for multiple purpuric lesions in the skin which are caused by extramedullary hematopoiesis, vascular processes, or neoplastic infiltrations. There are many underlying causes that need to be considered when a neonate presents with blueberry muffin syndrome. The differential diagnosis can be divided into neoplastic, dermatological, haematological and infectious (TORCHES) causes. Examples of these underlying causes are listed in Table 1 [3–5]. The blueberry muffin rash most commonly results from intrauterine infections, such as rubella and cytomegalovirus, and less commonly from a malignancy or hematologic disorder. To our knowledge, there is no validated diagnostic algorithm for neonates with a blueberry muffin rash [6]. We would suggest a multidisciplinary approach involving neonatologists, paediatricians, dermatologists, haematologists/oncologists and infectious disease specialists.

**Table 1:** Differential diagnosis of a blueberry muffin rash

Category	Diseases
Infectious	TORCHES^a^
Dermatologic	Haemangiomatosis, blue rubber bleb naevus syndrome, multifocal lymphangioendotheliomatosis, glomangiomatosis
Neoplastic	Congenital leukemia, neuroblastoma, congenital rhabdomyosarcoma, histiocytoses^b^
Hematologic	Blood dyscrasias^c^, disseminated intravascular coagulation, transient myeloproliferative disorder, mastocytosis, hereditary spherocytosis

^a^*TORCHES* Toxoplasmosis, Other (varicella-zoster virus, parvovirus B19, coxsackie virus, hepatitis B, Epstein Barr virus), Rubella, Cytomegalovirus (CMV), HErpes infections and Syphilis

^b^Langerhans cell histiocytosis, indeterminate histiocytosis, juvenile xanthogranuloma

^c^For example Rhesus incompatibility, twin-to-twin transfusion syndrome

After complete physical examination, a blood sample should be taken for blood count, blood film, coagulation and screening tests for infection. This includes maternal and infant serology and PCR for the infections included in the acronym TORCHES (see Table 1). Specialist review by a paediatric haematologist is recommended, as the initial features of leukemia may be subtle. In addition, skin biopsy is an important diagnostic investigation, which we strongly advise to perform in an early stage since the skin lesions might resolve within a couple of days, making diagnosis impossible. Skin biopsy might show for example congenital leukemia or histiocytic disorders. An ultrasound of the abdomen can be used to look for neuroblastoma.

In our case, the blueberry muffin rash was caused by ICH, which is extremely rare and can only be diagnosed by biopsy. Approximately 100 cases of ICH have been described. It usually occurs in adults and is of unknown etiology. Some cases appear to arise as an inflammatory reaction after for example scabies or a tick bite [7, 8]. A few cases concerned infants and to our knowledge, only one case in literature concerned a neonate [9]. Like our case, the skin lesion in this neonate spontaneously regressed, lacked Langerhans cells in histopathological examination and there were no signs of systemic disease. In contrast to our case, this neonate presented with a solitary lesion that was present at birth, while in our case multiple skin lesions developed shortly after birth.

ICH can present with a solitary or with multiple skin lesions, usually on the trunk and extremities [10]. Extracutaneous involvement is rare, but ICH lesions in the eye, in the bone and in the spleen have been reported [11–13]. The clinical course of ICH is diverse; it varies from solely cutaneous involvement with a self- limiting or chronic benign clinical course to severe systemic forms [14]. Early recognition of ICH and routine follow-up are important since multi-organ involvement and development of secondary hematological neoplasms, such as myeloid leukemias or low-grade B lymphomas, have been observed in adults [15, 16].

Multiple treatment options have been reported in the literature, varying from a wait- and-see policy, sometimes with additional corticosteroids, to systemic treatments and phototherapy [17]. No standard treatment regimen exists yet.

In our case, skin biopsy revealed proliferation of CD1a and S100 protein-positive dendritic cells, which were negative for Langerin (CD207). The lack of Langerin differentiates ICH from LCH [14]. Recurrent genetic alterations have been determined in many of the different types of histiocytosis [2, 18]. In our case, skin biopsy revealed a MAP2K1 mutation, which is known to be present in part of the patients with LCH [2]. The MAP2K1 mutation has been previously described in merely one case of ICH, which was a teenage boy [19]. Little is known about mutational profiles of patients with ICH and its impact on clinical progression.

In our patient, the purpuric eruptions dissolved spontaneously after 2 weeks. There was no systemic involvement. This disease course is similar to that of Congenital Self*-* Healing Langerhans Cell Histiocytosis or Hashimoto-Pritzker disease, a rare benign variant of LCH which typically appears at birth or in the neonatal period. It is characterized by multiple asymptomatic papulonodular lesions which resolve spontaneously within weeks to months. However, the presence of Langerin in the accumulating cells, distinguishes this LCH variant from ICH. Due to the rarity of ICH, little is known about the chances of remission and disease-free survival after spontaneous resolution of the lesions.

ICH is an exceptionally rare cause of blueberry muffin rash in neonates which can be diagnosed by skin biopsy. ICH is limited to the skin in most cases, but systemic development is possible. Therefore, it is essential to monitor these patients closely and perform routine follow-up. To this date, pathologic parameters to predict disease course have not been established.

## Data Availability

Not applicable.

## Abbreviations

ICH: Indeterminate Cell Histiocytosis
LCH: Langerhans Cell Histiocytosis
